# Post-discharge malaria chemoprevention (PMC) in Malawi: caregivers` acceptance and preferences with regard to delivery methods

**DOI:** 10.1186/s12913-018-3327-z

**Published:** 2018-07-11

**Authors:** Sarah Svege, Blessings Kaunda, Bjarne Robberstad, Thandile Nkosi-Gondwe, Kamija S. Phiri, Siri Lange

**Affiliations:** 1 Centre for International Health and Department of Global Public Health and Primary Care, University of Bergen, Bergen, Norway; 2 College of Medicine, University of Malawi, Blantyre, Malawi; 3 School of Public Health, University of Witwatersrand, Johannesburg, South Africa; 4 Chr. Michelsen Institute, Bergen, Norway; 5 Department of Health Promotion and Development, University of Bergen, Bergen, Norway

**Keywords:** Post-discharge malaria chemoprevention, Dihydroartemisinin-piperaquine, Community-based delivery, Facility-based delivery, Community health worker, Text message reminder, Public health card, Malawi

## Abstract

**Background:**

In malaria endemic countries of sub-Saharan Africa, many children develop severe anaemia due to previous and current malaria infections. After blood transfusions and antimalarial treatment at the hospital they are usually discharged without any follow-up. In the post-discharge period, these children may contract new malaria infections and develop rebound severe anaemia. A randomised placebo-controlled trial in Malawi showed 31% reduction in malaria- and anaemia-related deaths or hospital readmissions among children under 5 years of age given antimalarial drugs for 3 months post-discharge. Thus, post-discharge malaria chemoprevention (PMC) may provide substantial protection against malaria and anaemia in young children living in areas of high malaria transmission. A delivery implementation trial is currently being conducted in Malawi to determine the optimal strategy for PMC delivery. In the trial, PMC is delivered through community- or facility-based methods with or without the use of reminders via phone text message or visit from a Health Surveillance Assistant. This paper describes the acceptance of PMC among caregivers.

**Methods:**

From October to December 2016, 30 in-depth interviews and 5 focus group discussions were conducted with caregivers of children who recently completed the last treatment course in the trial. Views on the feasibility of various delivery methods and reminder strategies were collected. The interviews were transcribed verbatim, translated to English, and coded using the software programme NVivo.

**Results:**

Community-based delivery was perceived as more favourable than facility-based delivery due to easy home access to drugs and fewer financial concerns. Many caregivers reported lack of visits from Health Surveillance Assistants and preferred text message reminders sent directly to their phones rather than waiting on these visits. Positive attitudes towards active use of health cards for remembering treatment dates were especially evident. Additionally, caregivers shared positive experiences from participation in the programme and described dihydroartemisinin-piperaquine as a safe and effective antimalarial drug that improved the health and well-being of their children.

**Conclusions:**

Post-discharge malaria chemoprevention given to children under the age of 5 previously treated for severe anaemia is highly accepted among caregivers. Caregivers prefer community-based delivery with use of health cards as their primary tool of reference.

**Trial registration:**

NCT02721420 (February 13, 2016).

## Background

In sub-Saharan countries many children develop severe anaemia as a result of previous and current malaria infections. Standard treatment at the hospital is blood transfusion and a short treatment course of antimalarial drugs. After an episode of severe anaemia, the red blood cell production needs at least 6 weeks to recover. If children experience recrudescent malaria infections or contract new infections in this period, the haematological recovery is further prolonged and they are at risk of developing rebound severe anaemia [1].

No specific strategy of follow-up care for children in the post-discharge period has been established. In 2012, our research group conducted a randomised placebo-controlled trial in Malawi where children under 5 years of age with severe anaemia received post-discharge malaria chemoprevention (PMC) of artemether lumefantrine (AL) for 3 months [2]. By 6 months, 31% of deaths or hospital readmissions due to severe malaria or anaemia were prevented. These findings suggest that PMC may provide a substantial protection against malaria- and anaemia related morbidity and mortality among young children living in malaria endemic areas. Therefore, to inform policy and guide implementation, a delivery implementation trial is currently being conducted in Malawi to determine the optimal PMC delivery strategy (ClinicalTrials.gov NCT02721420).

Children enrolled in the trial are 4–59 months old, clinically stable with body weight of 5 kg or above, and have completed at least one blood transfusion. Study participants are required to receive the antimalarial drug dihydroartemisinin-piperaquine (DHP) at 2, 6 and 10 weeks post-discharge. Each treatment course lasts for 3 days and caregivers are instructed to dissolve the drugs in water before giving it to their children to drink. In previous intervention studies, DHP is reported to be a well-tolerated, long-acting and effective antimalarial drug with few serious adverse events [3–5].

Participants in the PMC trial are randomly allocated to one of five study arms, where the delivery strategies are either community- or facility-based (Table 1). In the community-based study arms (1 + 2 + 3) caregivers receive all drugs at the hospital before their children are discharged from the hospital. Caregivers are expected to remember treatment dates with the following aids:

**Table 1:** Study arms

Study arm	Community-based delivery	Facility-based delivery	Exclusive use of health card	Text message reminder	Visit from Health Surveillance Assistant	In-depth interviews (IDIs)	Focus group discussions (FGDs)
1	x		x			6	1 (*n* = 6)
2	x			x		6	1 (*n* = 8)
3	x				x	6	1 (*n* = 10)
4		x	x			6	1 (*n* = 6)
5		x		x		6	1 (*n* = 5)

**Arm 1.** Exclusive use of the child’s health card.

**Arm 2.** Text message reminder.

**Arm 3.** Visit from Health Surveillance Assistant.

In the facility-based study arms (4 + 5) caregivers are instructed to collect drugs at the hospital for each treatment course. They are expected to remember dates for drug collection with the following aids:

**Arm 4.** Exclusive use of the child’s health card.

**Arm 5.** Text message reminder.

The overarching objective is to determine which strategy is best suited for large-scale and long-time implementation of PMC in areas of high malaria transmission. This article presents a qualitative study on acceptance among caregivers. It is undertaken in parallel with the trial in Malawi, and explores views on PMC and the five proposed delivery mechanisms. Since PMC drugs are administered to children at home by caregivers, their awareness and acceptance towards the programme will greatly influence level of drug uptake.

### Qualitative study on acceptance and feasibility of PMC

No qualitative study has been published that investigated the acceptance of antimalarial drugs given as PMC. PMC is a malaria control strategy where antimalarial drugs are given as intermittent preventive treatment to children after discharge from the hospital. Intermittent preventive treatment (IPT) targets at-risk patients in areas of moderate-to-high malaria transmission, and evidence from previous clinical trials has resulted in recommendations from WHO to administer IPT to pregnant women (IPTp), infants (IPTi) and young children (IPTc) [6–8]. IPTp is delivered during antenatal care visits, whilst IPTi is administered to infants through the Expanded Programme of Immunisation (EPI) - and qualitative studies have shown high acceptance among providers and users in both programmes [9–11]. In the case of IPTc – or seasonal malaria chemoprevention (SMC) – no clear guidelines on treatment delivery have been developed. SMC targets a large group of children regardless of present malaria status, and is less applicable to local settings since these children have limited contact with the health system. However, previous clinical trials have shown high uptake of SMC when delivered by village health workers or volunteers at community level [12, 13].

Caregivers in study arms 2 and 5 in the delivery implementation trial receive a text message reminder when the treatment course is due. Network and mobile phone coverage is improving in sub-Saharan countries and the use of mobile and wireless technologies in health care (mHealth) has the potential to enhance access and adherence to treatment [14]. In paediatric malaria treatment, discordant evidence on the effectiveness of text message – or Short Message Service (SMS) – has been presented. A short, informative text message reminder has proven to increase level of adherence to antimalarial treatment among children [15] while other studies show little difference in adherence between SMS-intervention and control groups [16, 17]. A qualitative study from Kenya detected high level of acceptance among caregivers towards use of text-messages in malaria treatment of children [18]. Extensive phone ownership, network availability and literacy was reported as enabling factors, but the text message intervention was not applied to the local setting for continued investigation. Do caregivers in the PMC trial consider text message reminders to be applicable in a real-life rural Malawian context?

In study arm 3, caregivers are supposed to be visited by a community-based Health Surveillance Assistant (HSA) one or two days before each of the treatment courses. Previous studies on SMC have shown how involvement of community-based health workers positively affects drug uptake [12, 13]. The Malawian Ministry of Health hires HSAs to provide health promotion activities in rural and urban communities with populations of 1000 people or more. In prior qualitative studies, HSAs report a set of multifaceted challenges impairing their motivation and work performance [19, 20]. These challenges include supply shortages, heavy workload, lack of supervision and limited financial incentives. What are the perceptions of caregivers on the accountability of HSAs and the feasibility of reminder visits?

Adherence to preventive malaria treatment may be challenged by caregivers who question the value and necessity of giving drugs to children who seemingly appear healthy and have not been tested for malaria [21, 22]. Lack of preceding laboratory and screening assessments leads to uncertainty of treatment rationale - and caregivers may find it difficult to understand the importance of giving drugs as a preventive measure. How do caregivers in the PMC trial view the value and effectiveness of preventive malaria treatment?

## Methods

### Study setting

Study participants (*n* = 375) in the clinical parent study are recruited from 1480 villages in the catchment area of Zomba Central Hospital in the Zomba district of Malawi. The district has an estimated population of about 970,000 inhabitants. The majority of study participants reside in rural communities and their caregivers mainly perform subsistence farming or are involved in small-scale businesses (Table 2). In Malawi, primary school is grade 1–8 and secondary school is grade 9–12. 26% of women and 36% of men age 15–49 have at least some secondary education. In rural areas, 14% of women have never attended school, and only 1% attended more than secondary school compared with 12% in urban areas [23]. In our sub-study, 14.1% attended secondary school and the remaining 85.9% only had primary schooling. 53 out of 64 informants reported to be literate during enrolment to the main study. Malaria transmission in the Zomba district follows a perennial pattern with especially high transmission during the rainy season from November until April.

**Table 2:** Informant characterstics in % and n (total = 64)

Age	Marital status	Education (grade)	Number of children	Occupation	Hours to hospital
< 20 3.1(2)	Single 9.4 (6)	1–4 25 (16)	1–3 51.6 (33)	Farmer 68.8 (44)	1–2 65.6 (42)
20–30 54.7 (35)	Married 71.9 (46)	5–8 60.9 (39)	4–6 37.5 (24)	Trader 23.4 (15)	2.5–3.5 25 (16)
> 30 42.2 (27)	Divorced 18.7 (12)	9–12 14.1 (9)	7–9 10.9 (7)	Other 7.8 (5)	> 4 9.4 (6)

### Sample selection and data collection

Methodically, this study has a qualitative, exploratory and interpretative design [24]. From October to December 2016, 30 semi-structured in-depth interviews (IDIs) and 5 focus group discussions (FGDs) were conducted with caregivers of children in the clinical trial. Most participants in research interviews were female caregivers whose children recently completed the study.

Data collection tools were reviewed and refined by the first, second and last author before and during fieldwork. All interviews and focus groups were conducted in the local language Chichewa. Informants were purposively and strategically selected to ensure a wide range of urban-to-rural areas of residence, although the majority lived in rural areas. The objective of the qualitative research interviews was to elicit information on perceptions of PMC and the five delivery strategies. IDI guides consisted of open-ended questions on decision-making at household level, preferred method of drug delivery, economic concerns and views on preventive malaria treatment. Six caregivers in each study arm were selected to take part in IDIs. The second author (BK) conducted all IDIs in the homes or villages of caregivers. These locations are thought to be well-suited for disclosure of personal views on subjective and sensitive themes. SS took ethnographic field notes during all IDIs to describe interview setting and capture non-verbal observations. This supplementary data enhanced our understanding of the study context where beliefs, concerns and preferences of informants were shaped.

After finishing the IDIs, FGDs with 5–10 participants from each study arm were arranged. Participants were selected from the group of remaining caregivers that had not been included in IDIs. They were invited to attend FGDs through phone calls or in-person visits. Analysis of incoming data from IDIs generated interesting themes and trends which were further investigated during FGDs. Thus, results from in situ analysis of IDIs were used to sharpen topics for the FGD guide. FGDs had a special focus on comparing the benefits and barriers of the different delivery and reminder systems. All FGDs were conducted at Zomba Central Hospital by one facilitator and two note takers who are all native speakers of the local language. The hospital was an appropriate location for collecting perceptions on less sensitive topics, and logistically convenient considering the many villages represented within the groups. Participants received refreshments and travel reimbursement. The first author was present during all interviews and group discussions. IDIs and FGDs were recorded, transcribed verbatim and translated to English by three research assistants. Thorough quality checks on a random sample of transcripts were done prior to data analysis by comparing transcripts to audio recordings.

### Data analysis

English transcripts were imported into the coding software programme NVivo Version 11. Iterative and detailed transcript readings allowed a gradual familiarisation with and immersion into the data material. Thematic analysis followed the framework approach with pre-defined codes developed a priori on the basis of study aim, which were further supplemented by codes emerging from data analysis [25, 26]. Both deductive and inductive codes were included in a NVivo coding set. During flexible, recursive and interpretative analysis, text segments of interest were compared, contrasted and attached to appropriate codes. The data analysis was process-oriented and took place both during and after fieldwork through briefing sessions and discussions within the research team. This analytic path has facilitated extraction of meaning and discovery of regularities, similarities and divergence in the data material.

### Ethical considerations

Ethical approval for the PMC trial and its sub-studies was granted from the College of Medicine Research and Ethics Committee (COMREC) and the Pharmacies and Poisons Board of Malawi, as well as the Regional Committees for Medical and Health Research Ethics in Norway. During enrolment to the main study, a written informed consent was obtained from all caregivers of study participants. Additionally, a new written informed consent was collected from caregivers selected for qualitative interviews and group discussions. In cases of low literacy, informants would sign with a thumbprint or a witness signed on behalf of the informant. One consent form was kept by study staff, whilst another was given to the informant. All informants were masked with study ID numbers to ensure anonymity during IDIs and FGDs. Statements included in this article carry no personal identifiers. Data material is securely stored, and can only be accessed by study staff.

## Results

In this section we explore how caregivers consider benefits and barriers of community- and facility-based delivery of PMC (Table 3), and their thoughts on use of health card, text message reminders or HSA visits to remember treatment dates (Table 4).

**Table 3:** Reported benefits and barriers of facility- and community-based delivery of PMC

Main findings	Facility-based delivery	Community-based delivery
Benefits	* More contact with health personnel * Possibility of follow-up care at the hospital * Safe drug storage at hospital pharmacy	* Easy home access to drugs * Less financial concerns * Less worries related to care of children * More time for work tasks
Barriers	* Travel expenses * Extra work burden * Long travel distance * Challenging roads	* Perceived negative effect of longtime home drug storage on drug durability

**Table 4:** Reported benefits and barriers of text message reminder, visit from HSA and exclusive use of health card

Main findings	Text message reminder	Visit from HSA	Exclusive use of health card
Benefits	* Extra assurance * Reminder is sent directly to caregiver	* Face-to-face interaction * Assistance and counseling	* Active use of health card and memory * Strengthens self-reliance * Establishment of nearby reminder- and support system
Barriers	* Lack of phone * Prolonged delivery of text message via substitute phone owner * Network and electricity problems * Limited understanding of text message content due to low literacy	* HSA unreliability * Long distance between home of study participant and HSA * Reminder from the hospital goes via a HSA and is not transferred directly to the caregiver	* Challenges with remembering treatment dates * Limited understanding of health card content due to low literacy

### Facility- versus community-based delivery of PMC

During research interviews and discussions, the issue of transportation emerged as a potential barrier of facility-based delivery. Nearly all informants live in rural, hard-to-reach areas of the Zomba district. On their way to the hospital, they often walk in addition to utilising various modes of transport; like bicycle taxis and minibuses. Roads can be narrow, bumpy and in some places non-existent - and during the rainy season navigation is especially difficult. Nevertheless, according to informants the main hurdle in the facility-based study arms was lack of money to cover travel expenses. Many caregivers tackled this issue by doing extra labour or selling belongings. When pre-defined drug collection dates were near, they would strategically plan how to gather sufficient amounts of travel money:

*“The challenge I was facing was money. As you can see in this village – money is a problem. Sometimes you have to do some piecework in the farm or sell your chicken if you have one so that you can find some money for transport. If you do not have anything then you are in big trouble. So when you are supposed to be going to the hospital you get worried about how you are going to travel. You try to borrow money and if you have chickens you sell them to make the journey possible.”* (Mother in arm 5, IDI)

Some caregivers were forced to borrow money from friends and relatives, and had problems paying back:

*“Going to the hospital was a challenge to me because of the transportation expenses. Sometimes I had difficulties paying back the money I borrowed.”* (Mother in arm 4, IDI)

On drug collection dates, caregivers often used several hours of the day on travelling. For some, absence from home left work responsibilities untouched and children unsupervised. Many brought their youngest children along for the journey if no one could watch them at home. When roads were difficult and the weather was unpleasantly hot this journey became tiring for both the child and its caregiver. Still, some willingly brought their children along with the intention of seeking follow-up care at the hospital. They saw it as a chance to maintain ongoing dialogue with hospital staff and ask questions if necessary. Thus, a strength of facility-based drug delivery was increased follow-up care and continuing contact with health personnel. Additionally, a few caregivers emphasised the value of drugs being stored at the hospital. They believed drugs should not be kept at home for an extensive period of time since this may threaten durability and effectiveness of the drug, whereas a hospital pharmacy provides a safe space for drug storage:

*“It is better to collect medicine from the hospital considering that care for the medicine at the village may be hard with the houses we live in. Some of us stay in leaking houses so the medicine may even be soaked (…) and then we give it to the child. In this way you are giving a medicine that is not good to the child.”* (Father in arm 4, FGD)

Despite the benefits of strengthened follow-up care and safe drug storage, most informants in facility-based study arms spoke in favour of drug delivery through a community-based approach. They claimed that their concerns about the cost, time and risk of travelling to the hospital would mitigate if all drugs were collected at the hospital before discharge. Easy home access to drugs would ensure increased flexibility in matters of care and work:

*“We always face challenges when it comes to issues of transportation in this area. To get to the hospital we need money for transportation and we have to travel a long distance to get to the hospital. After collecting the drugs we have to travel the same distance back home. This is why I prefer collecting all the drugs at once.”* (Mother in arm 5, IDI)

*“Collecting all the drugs and having them at home is a good idea because when you do so you avoid a lot of things. If it happens that you do not have money for transportation on the day that you are supposed to go to the hospital to collect the drugs you don’t need to worry because you have everything that you need right there at home.”* (Mother in arm 2, FGD)

*“I was not finding any chance to do chores at home because I was waking up very early in the morning and there was no time to do anything else than going to the hospital to collect drugs.”* (Mother in arm 5, IDI)

A commitment to comply with treatment guidelines was evident among informants in both community- and facility-based study arms. Regardless of assigned delivery method, informants were dedicated and determined to follow instructions. Almost exclusively, they shared positive views on the trial drug and its effectiveness. Additionally, the appreciation and admiration towards health personnel at Zomba Central Hospital was apparent. Hence, economic constraints and other barriers did not overshadow their motivation to come for drug collection at the hospital. Still, most informants considered drug delivery at discharge less challenging both financially and personally. When asked to rank one method above the other, a clear majority preferred community-based delivery of PMC.

### Text message reminder versus visit from health surveillance assistant

In study arms 2 and 5, caregivers are supposed to be reminded via phone text message on days of drug administration or collection. Some informants described text messages as a “quick” and “easy” way of conveying reminders directly to caregivers. For them, it functioned as reassurance and encouragement on treatment dates:

*“I think the text message is a very good approach because sometimes you may get busy and forget.”* (Mother in arm 5, FGD)

Nevertheless, numerous barriers to the use of text message reminders were reported. A recurring theme was the challenge of phone usage. Informants persistently stressed how lack of electricity, inadequate charging services and network problems impede their ability to receive text messages. Another frequently expressed obstacle was phone availability. Several caregivers do not own a phone and reminders were sent to the phone of a relative, neighbour or friend instead. In these instances, the message was often conveyed via multiple intermediate carriers before finally arriving with the caregiver. Thus, the passing of information could be disturbed, prolonged and, in some cases, permanently halted:

*“Some of us do not have our own phones. We rely on other people’s phones to receive these reminders. Relying on phones may become a problem if the owner of that phone is not around and the message does not get to you in time.”* (Mother in arm 2, FGD)

*“My in-law received the reminder messages and would call one of the girls who lived right here in the community, and the girl would pass on the message to me. However, the girl who had the phone has now moved.”* (Mother in arm 2, IDI)

In study arm 3, caregivers are supposed to be visited by a Health Surveillance Assistant (HSA) when the treatment course is due. HSAs in Zomba district were instructed on their role in the PMC programme during briefing meetings held at local health centres before study initiation. The HSAs are supposed to act as message-carriers between health staff at hospital level and caregivers at community level.

Informants attached varying attributes to the HSAs during interviews and discussions. Some characterised them as lazy and negligent, whilst others shared an appreciation of HSAs and described them as helpful and generous. Caregivers in the HSA study arm acknowledged both benefits and barriers of this reminder strategy. They understood that the primary purpose of a HSA visit was to remind them on treatment dates, and some claimed that it also allowed exchange of questions and knowledge on malaria and other under-five childhood illnesses. The visit provided some of the caregivers with a sense of assurance that drugs were administered in a correct manner, and they addressed how a face-to-face interaction is prone to less misunderstanding than technology driven phone and text message communication:

*“When the HSA visit and we sit together it is possible to understand each other. You and I are sitting here and if there is something I do not understand I can ask “what did you say there?” but you cannot ask that through the phone, maybe I do not have airtime to call and ask for guidance.”* (Mother in arm 3, FGD)

*“A phone is both reliable and unreliable as well, like today they can send a message and remind me to give drugs to the child and the next day I will not be available maybe because a charger is broken or the battery is dead. There are electricity problems also and that is what we experience – so a phone is a bit hard to use and it will be better if the HSAs visit and remind us verbally.”* (Mother in arm 3, FGD)

Nonetheless, a widely reported barrier was the unreliability of HSAs. Many caregivers in the HSA study arm mentioned the unavailability of HSAs in their catchment area and lack of home visits by the designated HSA. While some informants reported being visited by the HSA either one or three times during the study period, most stated that they received no visits but still remembered to give medicine to their children:

*“The HSA did not visit me even once. However, I gave my child the drugs according to the instructions in the book (health card) and I did not miss (…) I felt like the HSA neglected us because he did not even call me, so I just calculated the dates and numbers in my head.”* (Mother in arm 3, FGD)

*“Since the medicine was already here, I just took it and gave it to the child considering that the HSA who is working may be busy with other things so if we are waiting to be told we will end up not giving the medicine to the child.”* (Mother in arm 3, IDI)

When informants across all study arms were asked to compare the two reminder strategies, the majority ranked text messages as their preferred method. They wanted reminders to be sent directly to them instead of going through a HSA:

*“I would recommend the mobile phone approach because in this way the reminder will come directly to me.”* (Mother in arm 3, IDI)

Although many informants were positive towards the use of text message reminders, the success of this model depends on whether conditions are favourable and caregivers own well-functioning phones. In theory, text messages are quick and efficient sources of information – but experiences from caregivers suggest that a tech-dependent reminder system in the current rural Malawian setting is difficult:

*“I would prefer using the phone because it is quicker (..) but it is just that the battery on my phone has a problem so it is unreachable.”* (Mother in arm 3, IDI)

### Reminders versus relying on health card and memory

Every child in the trial has a national public health card where dates for PMC treatment courses are written. In study arms 1 and 4, caregivers receive verbal instructions at the hospital and are supposed to remember the dates themselves. Caregivers in these study arms repeatedly checked the health card and they often asked a spouse, relative or friend to remind them when treatment dates were approaching. Thus, they created their own reminder- and support system of people with the child’s best interest at heart:

*“On one of the days I nearly forgot to give the drug to the child because I was busy but my husband reminded me.”* (Mother in arm 4, IDI)

During IDIs and FGDs, caregivers in all five study arms were informed on the various delivery strategies and asked to compare and decide their preferred method. The great majority shared positive attitudes towards use of a health card to remember treatment dates, and a high number of informants insisted they could be self-dependent and administer drugs to their children without reminders:

*“The most recommendable approach would be the one where all information is written in the health card. In that way I would always remind myself by constantly checking the dates.”* (Mother in arm 3, IDI)

Caregivers claimed that information in the health card was easily accessed due to its constant presence at home, and since it contains important health-related information they would usually keep it in a safe place. Many caregivers who were supposed to receive reminders reported that they would administer drugs according to health card instructions even in the absence of reminders. Hence, active use of the health card evoked an inherent sense of independence and self-reliance among caregivers:

*“The health card is a good approach because when you have all the information in the health card you don’t have to rely on someone to remind you about the dates. Sometimes you may wait for a text message which may never come and this becomes a problem to your child.”* (Mother in arm 2, FGD)

A few caregivers reported low literacy as the only potential challenge to the use of health card. In their opinion, those who had never gone to school or spent a few years in primary school would have difficulties understanding content in both the health card and text messages. Nevertheless, many informants argued that it was easy for those with limited primary school education to find a family member or friend who could read the health information for them. The many positive perceptions on use of health card and memory show how caregivers want to take responsibility for the collection and administration of drugs to their children:

*“I would recommend the method where I can remind myself about the time to give the drug to the child. I don’t think waiting for someone to remind me would be recommendable because they may one day forget and the child will not get the drug that day (…) I can’t get used to the fact that someone has to come to remind me about the dates of giving the drug to the child – I have to do that on my own.”* (Mother in arm 4, IDI)

*“The best is to write in the book (health card) because HSAs have their own problems to think about. Maybe on the day they were supposed to remind you, they have gone for training. This means that you will not be reminded. While with the text messages – the message may be sent but at that time the phone is off and you will see it after two days. With the book (health card) you can easily refer to it anytime.”* (Father in arm 4, FGD)

### Self-reported compliance and views on preventive malaria treatment

A high degree of self-reported compliance was discovered among informants. Only a few stated that they missed a treatment date, and several factors might have contributed to the high level of compliance. Caregivers had recently experienced how severe anaemia detrimentally impaired the health and well-being of their children. After this experience they are familiar with the symptoms, severity and dangers of the disease, and the past event generated a feeling of fear which functioned as a driving force for treatment compliance:

*“I had the memory of how my child was sick and the stage he reached. So I made it a point not to belittle the medicine, but to give them to him every day until the dosage was finished so that I could see how helpful the medicine was.”* (Mother in arm 1, IDI)

During enrolment to the trial, health staff instructed caregivers on drug administration and underlined the importance of following treatment guidelines. Most informants described how they would give the drugs in the morning at the same time for three consecutive days. Also, they fed their children before giving the drugs. These findings indicate that caregivers had a good understanding of information provided at the hospital. In addition, informants claimed that a facilitating factor for drug adherence was regular follow-up visits. A member of study staff visited the participant’s home to do pill counts after each treatment course. The expectation of these encounters could have unrealistically enhanced treatment adherence. Thus, context and circumstances of the trial setting might have contributed to the high level of acceptance and self-reported compliance among caregivers.

Overall, informants were positive towards participation in the programme. The DHP drug was considered safe and effective due to its observed impact on the health status and well-being of study participants. Caregivers reported improvement in the health, appetite, mood, energy level and appearance of their children. When asked about potential barriers to participation, they mentioned transport costs and logistic difficulties in facility-based delivery and the risk of longtime home drug storage in community-based delivery. Consequently, some suggested storing and distributing drugs at local health facilities instead of the hospital:

*“It is important that antimalarials are given to children when they have been discharged from the hospital, but they should not let the caregivers travel all the way to the hospital to collect the drug for the sake of those who live far away (…) It is better to have the drugs at the nearest health facility where they can be cared for and easily accessed.”* (Mother in arm 5, IDI)

Scepticism and superstition among neighbours and other village members was also reported by a few informants as complicating factors for participation:

*“People should continue to give the drugs despite of what other people say. Some people insinuate that if your child is taking the antimalarial drugs it means they are HIV-positive. I did not care what people said because I knew my child needed help and the drug had nothing to do with HIV.”* (Mother in arm 5, IDI)

*“When I went home after being discharged from the hospital, some of the people in the village told me that I had involved myself into Satanism by enrolling my child in this programme. But I took the document which I was given at the hospital and gave it to them as evidence for what I was really involved in. I also explained that my child was sick and right now he was put on a dosage of antimalarial drugs so therefore I will be going to the hospital to collect the drugs.”* (Mother in arm 5, FGD)

In some cases, resistance among community members was rooted in limited understanding of the necessity of giving drugs to children who appeared healthy and were already discharged from the hospital:

*“There are some women who are afraid to put their children on more antimalarials simply because they have already been treated at the hospital and they are doing much better. However, they do not know that this is something that can be of much help to their children.”* (Mother in arm 2, FGD)

After witnessing the beneficial effects of the drugs themselves, caregivers tried to abolish other people’s distrust by showcasing the health improvement of their children, and by sharing their acquired knowledge on the value of preventive malaria treatment:

*“I would tell other caregivers not to be discouraged if they are asked to take part in the programme (…) they should not be afraid or think that they will be given a difficult or bad medicine. All they (health staff) want is for your child to be well (…) my child was part of this and she has strength now.”* (Mother in arm 4, FGD)

*“Since we don’t know where the illness is hiding the antimalarial drugs are a good thing because they act like a shield and are really protecting the child.”* (Mother in arm 2, IDI)

Another suggested strategy for battling resistance, reluctance and rumours was sensitisation meetings directed by health staff or HSAs at community level. Informants stated that such meetings could increase awareness and acceptance of the programme, and several informants highlighted the importance of including village heads since they usually have great influence and authority in their respective communities. Additionally, many noted that they – as caregivers of children in the programme - should function as role models and share their experiences with other caregivers:

*“I can be of help in this programme because I have been part of it so I can encourage my friends after having experienced the goodness of malaria prevention.”* (Mother in arm 5, IDI)

*“I can help my fellow women with an orientation on how the drug should be given to the child. I would also use my situation as an example and share my experiences on how I gave this drug to my child.”* (Mother in arm 2, FGD)

## Discussion

In contrast to IPTp, IPTi and SMC (IPTc), the PMC (IPTpd) programme is directed towards a smaller population of seriously ill patients. These patients have already established connections to health staff after a recent hospital admission, and PMC is therefore perceived as a “natural” continuation or follow-up treatment and results from this qualitative study show that it is well-accepted among caregivers. Even though their children seem healthy, caregivers in the programme understand the value of giving preventive malaria drugs in the post-discharge period. The fear of reliving past experiences where their children suffered from severe malarial anaemia seems to function as a driving factor for treatment adherence. Also, caregivers share their experiences from the programme with community members and thus contribute to increased general acceptance towards PMC. Our findings support a previous study which showed high caregiver acceptance of DHP in malaria treatment of young children in Malawi [27].

Focus and dedication towards the programme is easily detected among caregivers and other individuals closely related to the child. Conversely, those with less emotional attachment to the child are likely to not have the same degree of interest in the child’s health improvement and treatment dates. For this reason, caregivers should not depend entirely on relatives, neighbours or friends to remind them. This may pose a threat to the accuracy and timeliness of drug administration. Neither should they rely completely on a text message which they may not receive due to phone problems, or a HSA visit which they may not receive for other reasons. Depending heavily on reminders can hamper their own efforts to remember treatment dates and threaten their sense of responsibility in the programme. Enabling and blocking factors of both reminder models have been presented, and the main barrier in the HSA study arm was lack of reminder visits. This might be explained by the heavy work load of HSAs which led them to either forget or choose to not prioritise the visits. HSAs are supposed to receive a text message with instructions from study staff, and faults in this communication pathway can also explain the lack of visits. Additionally, the willingness of HSAs to adhere is possibly limited by the fact that they receive no additional remuneration for doing reminder visits.

Furthermore, we see that most caregivers prefer text message reminders rather than HSA visits. Preferably, the reminder should be sent directly to their personal phones. If this is not possible they should live in close proximity to the substitute phone owner. Those who struggle with remembering treatment dates may find the reminder imperative, while the ones already aware of the dates regard it as a convenient confirmation of less importance. Hence, caregivers *do* benefit from the support of reminders - but they also benefit substantially from investing energy and effort into active use of the health card in order to remember treatment dates on their own. Our most surprising finding is the high acceptance towards exclusive use of the conventional, old-school health card with recorded treatment dates and medical history. Among informants, this card is regarded as a valuable treasure-like object that must be taken good care of.

### Study limitations

Data collected during qualitative research interviews and discussions is prone to social desirability bias [28]. Participants may have a desire to respond positively in order to please interviewers or secure future support from hospital staff. Also, responses might have been affected by recall bias although most caregivers had participated in the programme recently.

There was a mix of male and female participants in 3 of 5 FGDs, while most IDIs only included female caregivers. A greater inclusion of male caregivers could have expanded the range of viewpoints in the data material. The number of IDIs and FGDs conducted in each study arm was predetermined based on available time and financial resources. We did not know in advance whether a point of data saturation would be reached, but daily briefing sessions and data analysis performed during fieldwork assured us that saturation was achieved and no additional interviews were added.

The level of acceptance and awareness towards PMC may be higher in this trial compared to a non-trial setting with limited follow-up and less instructions from health staff. Our results are drawn exclusively from qualitative research interviews and discussions, and it remains to see whether findings from the main study will mirror the high degree of self-reported compliance found in this study. Other ongoing sub-studies will bring forth evidence on cost-effectiveness, drug uptake levels and clinical outcomes.

## Conclusions

Our findings reveal a high degree of acceptance towards malaria chemoprevention given as post-discharge management of children treated for severe anaemia. Receiving all drugs before discharge is perceived as more acceptable than collecting drugs for each treatment course at the hospital. Text messages are viewed as a more feasible strategy for reminding caregivers of treatment dates than visits from Health Surveillance Assistants. Nevertheless, the success of both reminder systems is challenged by complicating factors in the rural Malawian context.

Our findings indicate that the national health card is the most secure, reliable and easily accessible tool for health related information. It is viewed as the unwavering source of information on treatment dates and the majority of caregivers use it as their primary tool of reference. Exclusive use of the health card has the advantage of not adding extra costs to caregivers or the health system. In a possible future implementation of post-discharge malaria chemoprevention (PMC), caregivers should be informed on the importance of their commitment for the success of this treatment. If caregivers are conscious about their pivotal role in the programme it will strengthen their willingness and motivation to comply.

## Abbreviations

AL: Artemether lumefantrine
DHP: Dihydroartemisinin-piperaquine
EPI: Expanded programme on immunisation
FGD: Focus group discussion
HSA: Health Surveillance Assistant
IDI: In-depth interview
IPT: Intermittent preventive treatment
IPTc: Intermittent preventive treatment in children
IPTi: Intermittent preventive treatment in infancy
IPTp: Intermittent preventive treatment in pregnancy
IPTpd: Intermittent preventive treatment post-discharge
PMC: Post-discharge malaria chemoprevention
SMC: Seasonal malaria chemoprevention
SMS: Short-message service
